# Hypoxia causes reductions in birth weight by altering maternal glucose and lipid metabolism

**DOI:** 10.1038/s41598-018-31908-2

**Published:** 2018-09-11

**Authors:** Jenni Määttä, Niina Sissala, Elitsa Y. Dimova, Raisa Serpi, Lorna G. Moore, Peppi Koivunen

**Affiliations:** 1 Biocenter Oulu, Faculty of Biochemistry and Molecular Medicine, Oulu Center for Cell-Matrix Research, University of Oulu, Oulu, Finland; 2 Department of Obstetrics & Gynecology, University of Colorado Denver School of Medicine, Aurora, CO United States

**Keywords:** Homeostasis, Reproductive biology

## Abstract

Hypoxia of residence at high altitude (>2500 m) decreases birth weight. Lower birth weight associates with infant mortality and morbidity and increased susceptibility to later-in-life cardiovascular and metabolic diseases. We sought to determine the effects of hypoxia on maternal glucose and lipid metabolism and their contributions to fetal weight. C57BL6/NCrl mice, housed throughout gestation in normobaric hypoxia (15% oxygen) or normoxia, were studied at mid (E9.5) or late gestation (E17.5). Fetal weight at E17.5 was 7% lower under hypoxia than normoxia. The hypoxic compared with normoxic dams had ~20% less gonadal white adipose tissue at mid and late gestation. The hypoxic dams had better glucose tolerance and insulin sensitivity compared with normoxic dams and failed to develop insulin resistance in late gestation. They also had increased glucagon levels. Glucose uptake to most maternal tissues was ~2-fold greater in the hypoxic than normoxic dams. The alterations in maternal metabolism in hypoxia were associated with upregulation of hypoxia-inducible factor (HIF) target genes that serve, in turn, to increase glycolytic metabolism. We conclude that environmental hypoxia alters maternal metabolism by upregulating the HIF-pathway, and suggest that interventions that antagonize such changes in metabolism in high-altitude pregnancy may be helpful for preserving fetal growth.

## Introduction

Living at high altitude reduced oxygen availability decreases birth weight by 100 g/1000 m^1–3^ and is associated with increased infant mortality. Oxygen scarcity or hypoxia activates the cellular hypoxia-response pathway^4^. Acutely, hypoxia stimulates the sympathetic nervous system and carotid chemoreceptors to raise ventilation^5^. A major adaptation, however, occurs at the transcriptional level and is governed by the hypoxia-inducible factor (HIF)^4^ and HIF prolyl 4-hydroxylases (HIF-P4Hs), enzymes that act as cellular oxygen sensors^4,6,7^. When oxygen is available, HIF-P4Hs target HIFα subunits for proteasomal degradation whereas under hypoxia, their activity is inhibited, HIFα becomes stabilized and forms a transcriptionally active αβ dimer^4^. There are more than 300 genes that contain a hypoxia response element to which HIF binds to promote transcription. Products of the HIF target genes balance oxygen supply and demand by increasing oxygen delivery and/or reducing its usage. Some key adaptations are the induction of erythropoiesis and angiogenesis by upregulation of erythropoietin and vascular endothelial growth factor, respectively, and the shift in energy metabolism to increase oxygen-independent glycolytic metabolism and decrease oxidative phosphorylation (OXPHOS)^4,8^. HIF also upregulates glucose transporters (GLUTs) and the key enzymes of glycolysis, such as phosphofructokinase (PFK), and pyruvate dehydrogenase kinase (PDK) that inactivates pyruvate dehydrogenase and therefore prevents pyruvate entry to the Krebs cycle and OXPHOS^8^.

Maternal metabolism undergoes major changes during pregnancy^9–12^. The first two trimesters are characterized by an anabolic phase while the last trimester is catabolic. During the anabolic phase, lipid deposition to maternal tissues is increased, being facilitated by enhanced *de novo* lipogenesis. In the catabolic phase, the fat deposits are broken down to support the accelerated fetal growth which is further supported by the increased maternal hepatic gluconeogenesis and ketogenesis. The last phase of pregnancy is characterized by maternal hyperlipidemia and progressive insulin resistance, with the latter serving to direct glucose to fetal rather than maternal tissues.

While lower birth weight increases fetal and perinatal mortality and morbidity, it also predisposes to metabolic dysfunction at adult age^13^. Activation of the hypoxia response by genetic or pharmacologic inhibition of HIF-P4Hs and the concomitant HIF stabilization has been shown to have beneficial effects on metabolism in non-pregnant mice^14–18^. These include protection against obesity, lower adipose tissue inflammation, lower serum cholesterol levels, improved glucose tolerance and protection against insulin resistance^14,15^.

We set out here to study the effect of environmental hypoxia on maternal glucose and lipid metabolism during pregnancy and fetal weight in C57BL6/NCrl dams. Our data show that dams housed under hypoxic conditions have altered glucose and lipid metabolism compared to normoxic dams, and that these alterations are associated with the lower birth weight in hypoxia. These findings suggest that therapeutics or interventions that antagonize such changes in metabolism may have potential for increasing birth weight at high altitude and for ameliorating mortality and morbidity risk during early or later in life.

## Results

### Hypoxia reduced birth weight and maternal weight gain

**Figure 1:**
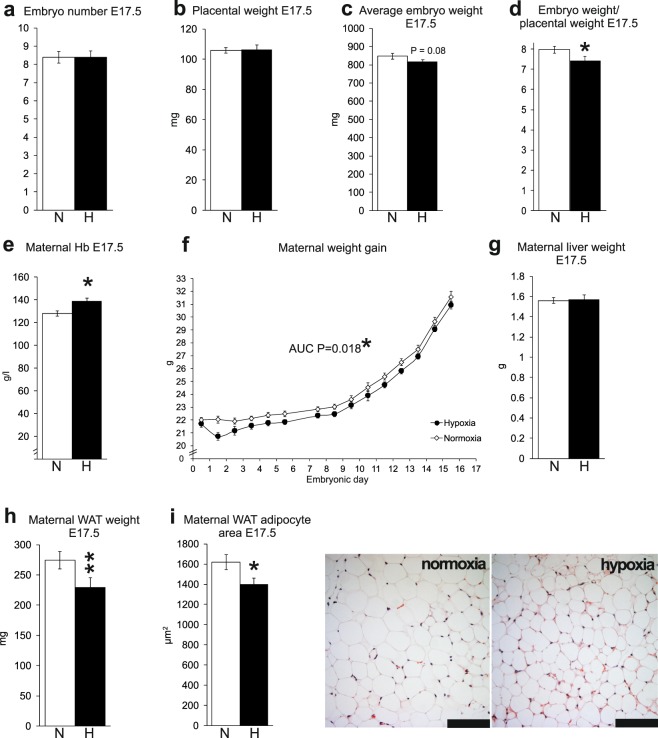
Hypoxic conditions reduced maternal weight gain, maternal white adipose tissue (WAT) and fetal birth weight. (**a**) Embryo number at E17.5 n = 66 (N), n = 45 (H). (**b**) Placental weight at E17.5 n = 43 (N), n = 25 (H). (**c**) Average embryo weight at E17.5 n = 45 (N), n = 30 (H). (**d**) Ratio of embryo weight to placental weight at E17.5. n = 23 (N), n = 10 (H). (**e**) Maternal haemoglobin at E17.5 n = 9 (N), n = 6 (H). (**f**) Gestational weight gain of dams n = 46 (N), n = 62 (H). (**g**) Maternal liver weight at E17.5. n = 43 (N), n = 26 (H). (**h**) Maternal gonadal WAT weight at E17.5 n = 67 (N), n = 46 (H). (**i**) WAT adipocyte area at E17.5 and corresponding histology, scale bar 100 µm n = 4 (N), n = 4 (H). N = normoxia, H = hypoxia (15% O_2_). Data are mean ± SEM. *P < 0.05, **P < 0.01. In (**c**) and (**d**) one-tailed t test was used.

C57BL6/NCrl dams were housed throughout gestation in normobaric hypoxia (15% oxygen which equals oxygen tension at 2700 m) or in normoxia (21% oxygen). The dams were fed *ad libitum* and sacrificed at E9.5 (mid pregnancy) or E17.5 (late pregnancy). There was no difference in embryo number or placental weight at E17.5 between the hypoxic and normoxic dams (Fig. 1a,b). As expected, the embryo weight was reduced under hypoxia (Fig. 1c,d). The average embryo weight at E17.5 under hypoxia was 4% less (Fig. 1c) and the ratio of embryo weight to placental weight was 7% less than those in normoxia (Fig. 1d, P < 0.05). Maternal hemoglobin (Hb) levels were significantly higher under hypoxia than normoxia at E17.5 (Fig. 1e), indicative of the hypoxia response being activated in the former^4^. The maternal weight gain was reduced under hypoxia during the whole gestational period compared with normoxia as the result, principally, of reductions in body weight during the first days of hypoxic exposure (Fig. 1f, area under the curve (AUC) P < 0.05). While there was no difference in the liver weights between the normoxic and hypoxic dams at E17.5 (Fig. 1g), the amount of gonadal white adipose tissue (WAT) was 16% lower and gonadal WAT adipocyte size was 14% smaller in the hypoxic group compared with normoxia (Fig. 1h,i, P < 0.01 and P < 0.05, respectively). Importantly, the amount of gonadal WAT correlated positively with embryonic weight (r = 0.32, P < 0.01).

### Reduced maternal adiposity under hypoxia contributed to impaired catabolism in late pregnancy

**Figure 2:**
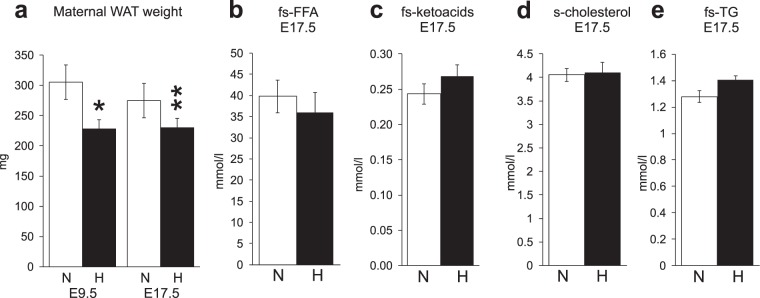
Exposure to hypoxic conditions during pregnancy reduced gestational anabolic metabolism, resulting in lower adipose tissue weight. (**a**) Maternal gonadal white adipose tissue (WAT) weight E9.5 and E17.5 in normoxic and hypoxic conditions. E9.5 n = 12 (N), n = 9 (H); E17.5 n = 67 (N), n = 46 (H). (**b**) Maternal fasting serum free fatty acids (FFA) at E17.5 n = 9 (N), n = 6 (H). (**c**) Maternal fasting serum ketoacids at E17.5 n = 9 (N), n = 6 (H). (**d**) Maternal serum cholesterol at E17.5 n = 18 (N), n = 13 (H). (**e**) Maternal fasting serum triglycerides (TG) at E17.5 n = 18 (N), n = 13 (H). N = normoxia, H = hypoxia (15% O_2_). All data are mean ± SEM. *P < 0.05, **P < 0.01.

There was a 25% reduction in the amount of gonadal WAT in the hypoxic compared to normoxic dams at E9.5 (Fig. 2a, P < 0.05). When the change in gonadal WAT weight from E9.5 to 17.5 was measured it appeared to be decreased under normoxia (−11%, P = 0.29) whereas no difference was detected in the hypoxic dams (+1%, P = 0.93) (Fig. 2a), suggesting that the greater amount of gonadal WAT at mid-gestation in the normoxic group constituted a reserve that was drawn down during late gestation but that such a reserve was not generated for the hypoxic group. Moreover, the level of fasted serum free fatty acids at E17.5 was 11% higher in normoxia compared to hypoxia (Fig. 2b). Although this difference did not reach significance it was suggestive of higher lipolytic capacity in the normoxic dams. The level of ketoacids in the urine in the normoxic dams was also significantly higher than that present in hypoxia at E17.5 (Table 1), indicative of the increased lipolysis and likely contributing to the lower pH in the former (Table 1). No significant difference in the level of serum ketoacids, cholesterol or triglycerides were detected between the normoxic and hypoxic dams at E17.5 (Fig. 2c–e).

**Table 1:** Urine dipstick analysis of E17.5 dams housed in normoxia or hypoxia. n = 20 (N), n = 13 (H).

	Normoxia	Hypoxia	P
Ketoacids (mmol/L)	4 ± 1	1 ± 0	0.0006
pH	6 ± 0	7 ± 0	0.035
Glucose (mmol/L)	0.0 ± 0.0	0.0 ± 0.0	—
Protein (g/L)	0.4 ± 0.1	0.2 ± 0.0	0.012

All values shown are means ± SEM.

### Hypoxia improved glucose tolerance and reduced insulin resistance

**Figure 3:**
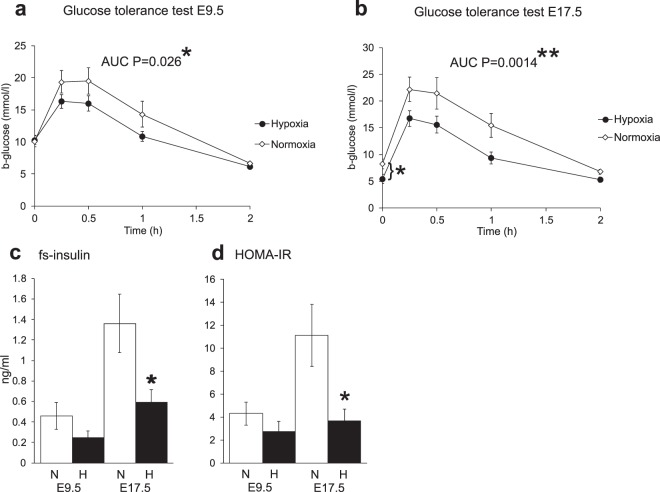
Maternal glucose tolerance and insulin sensitivity are improved under hypoxic conditions. (**a**) Glucose tolerance test (GTT) at E9.5 n = 11 (N), n = 11 (H). (**b**) GTT at E17.5 n = 8 (N), n = 7 (H). (**c**) Fasting serum insulin at E9.5 n = 9 (N), n = 6 (H) and E17.5 n = 15 (N), n = 8 (H). (**d**) Homeostatic model assessment for insulin resistance (HOMA-IR) at E9.5 n = 9 (N), n = 6 (H) and E17.5 n = 15 (N), n = 8 (H). N = normoxia, H = hypoxia (15% O_2_). All data are mean ± SEM. *P < 0.05, **P < 0.01. P values in (**c**) and (**d**) were calculated from the log transformed values.

Dams housed under hypoxia had improved glucose tolerance at E9.5 compared to the normoxic dams as shown by the results of the glucose tolerance test (GTT) (Fig. 3a, AUC P < 0.05). There was no difference in the fasting blood glucose levels between normoxia and hypoxia at E9.5 (Fig. 3a, time point 0 h). In accord with the reported decline in maternal glucose level in late pregnancy^9–12^, fasting blood glucose levels declined from E9.5 to E17.5, with the decline being significantly greater in the hypoxic than normoxic group at E17.5 (Fig. 3b, time point 0 h). The difference in GTT curves between the intervention groups was even larger at E17.5 than E9.5 (Fig. 3a,b). The hypoxic dams had lower serum fasting insulin levels (Fig. 3c) and a lower homeostatic model assessment-insulin resistance (HOMA-IR) score at E9.5 (Fig. 3d), although these differences did not reach statistical significance. In the normoxic dams, the greater increase in GTT from E9.5 to E17.5 was accompanied by a ~3-fold increase in fasting insulin levels (0.46 vs 1.36 ng/ml, P < 0.05) (Fig. 3c) and the HOMA-IR scores (4.3 vs 11.1, P < 0.05) (Fig. 3d). In contrast, fasting insulin levels only doubled in hypoxic dams from E9.5 to E17.5 (0.25 vs 0.60 ng/ml, P = 0.06) (Fig. 3c) and their HOMA-IR scores did not differ (2.8 vs 3.7, P = 0.52) (Fig. 3d), suggesting that their greater glucose tolerance at E17.5 was due to a failure to increase their insulin resistance in late pregnancy.

### Increased glucagon levels under hypoxia

**Figure 4:**
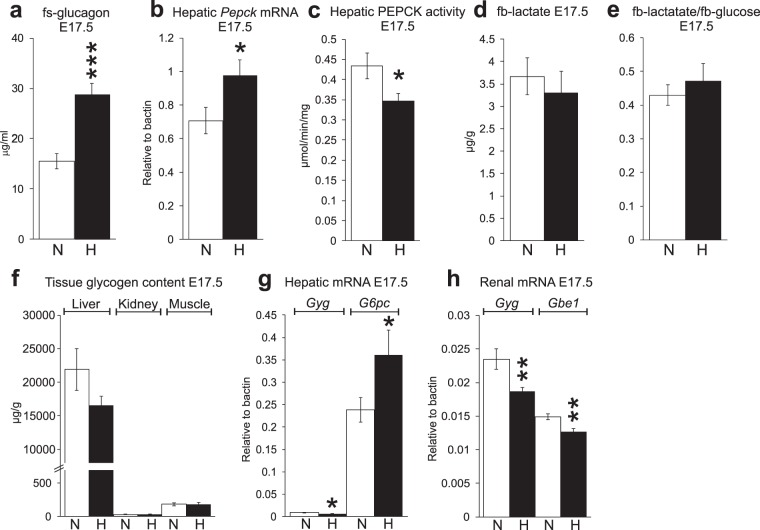
Hypoxia resulted in increased maternal serum glucagon levels and reduced gluconeogenesis and glycogenesis. (**a**) Maternal fasting serum glucagon at E17.5. (**b**) Maternal hepatic *Pepck* mRNA levels at E17.5 n = 8 (N), n = 9 (H). (**c**) Maternal hepatic PEPCK activity at E17.5 n = 6 (N), n = 6 (H). (**d**) Maternal fasting blood lactate levels at E17.5 n = 27 (N), n = 19 (H). (**e**) Maternal fasting blood lactate/fasting blood glucose ratio at E17.5 n = 27 (N), n = 19 (H). (**f**) Maternal tissue glycogen content in liver, kidney and muscle at E17.5. Liver n = 9 (N), n = 6 (H), kidney n = 9 (N), n = 6 (H), muscle n = 7 (N), n = 6 (H). (**g**) Maternal hepatic *Gyg* and *G6pc* mRNA levels at E17.5 n = 7–8 (N), n = 9 (H). (**h**) Maternal renal *Gyg* and *Gbe1* mRNA levels at E17.5 n = 7 (N), n = 8–9 (H). Gbe1 = glycogen branching enzyme 1, G6pc = glucose-6-phosphatase, Gyg = glycogenin, Pepck = phosphoenolpyruvate carboxykinase. N = normoxia, H = hypoxia (15% O_2_). All data are mean ± SEM. *P < 0.05, **P < 0.01, ***P < 0.001.

In late pregnancy, in addition to increased insulin resistance the supply of glucose for fetal growth is derived from increased lipolysis and ketogenesis, which in turn are supported by enhanced gluconeogenesis and glycogenolysis^10–12^. The levels of glucagon, the key hormone inducing gluconeogenesis and glycogenolysis, were 2-fold higher in the hypoxic dams at E17.5 compared with normoxia (Fig. 4a, P < 0.001). This may stem from the lower insulin levels in the hypoxia dams (Fig. 3c), insulin being a central inhibitor of glucagon secretion. The higher glucagon levels in hypoxia were associated with increased mRNA expression levels of hepatic *Phospoenolpyruvate carboxykinase* (*Pepck*) (Fig. 4b). However, when maternal hepatic PEPCK catalytic activity was measured at E17.5, the hypoxic compared with the normoxic livers had significantly lower activity (Fig. 4c, P < 0.05). The major substrates for gluconeogenesis in mammals are glucogenic amino acids, lactate and glycerol, with glycerol bypassing PEPCK in gluconeogenesis. There were no significant differences in serum fasting lactate levels (Fig. 4d) or the lactate to glucose ratio between the normoxic and hypoxic dams at E17.5 (Fig. 4e). These data would suggest that if the reduced hepatic PEPCK activity under hypoxia was due to scarcity of gluconeogenic substrates, reduced amount of glucogenic amino acids was likely responsible.

We next determined the amount of glycogen in the key gluconeogenetic tissues, liver, kidney and skeletal muscle at E17.5. The glycogen level in liver was 25% lower in the hypoxic compared with normoxic dams (P = 0.16) whereas its levels were similar in kidney or skeletal muscle tissues (Fig. 4f). The mRNA levels for *Glycogenin* (*Gyg*) were significantly downregulated in the hypoxic dam liver and kidney (Fig. 4g,h), as was that for *Glycogen branching enzyme 1* (*Gbe1*) in kidney (Fig. 4h), whereas the mRNA level for *Glucose-6-phosphatase* (*G6pc*) was upregulated in the hypoxic liver (Fig. 4g). Altogether these data suggest downregulation of glycogenesis and upregulation of glycogenolysis in hypoxia compared with normoxia.

### Increased uptake of glucose to maternal tissues correlated with upregulation of HIF target genes

**Figure 5:**
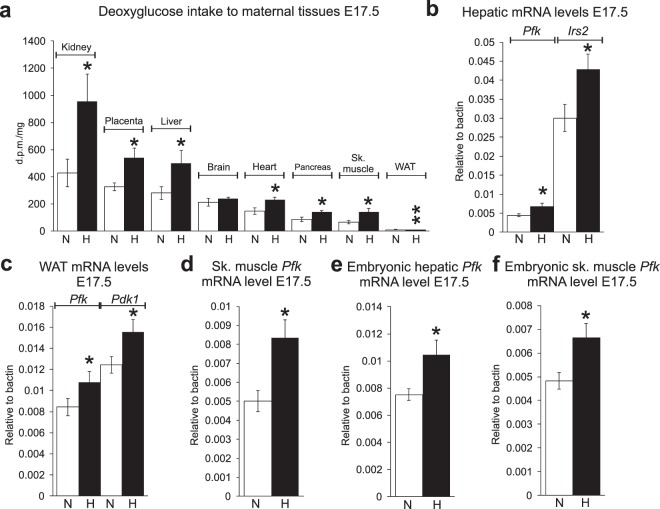
Hypoxia increased maternal glucose uptake and mRNA levels in several tissues. (**a**) Uptake of deoxyglucose into maternal kidney, placenta, liver, brain, heart, pancreas, skeletal (Sk.) muscle, gonadal white adipose tissue (WAT) in 4 h fasted dams. n = 8 (N), n = 7 (H). (**b**) Maternal hepatic *Pfk* and *Irs2* mRNA levels at E17.5 n = 8 (N), n = 9 (H). (**c**) Maternal WAT *Pfk* and *Pdk1* mRNA levels at E17.5 n = 7 (N), n = 9 (H). (**d**) Maternal skeletal muscle *Pfk* mRNA level at E17.5 n = 7 (N), n = 9 (H). (**e**) Embryonic liver *Pfk* mRNA level at E17.5 n = 7 (N), n = 9 (H). (**f**) Embryonic skeletal muscle *Pfk* mRNA level at E17.5 n = 6 (N), n = 8 (H). d.p.m., disintegrations per min. Irs2 = insulin receptor substrate 2, Pdk1 = pyruvate dehydrogenase kinase 1, Pfk = phosphofructokinase. All data are mean ± SEM. *P < 0.05, **P < 0.01.

To gain a deeper understanding of the differences in metabolism between the normoxic vs. hypoxic dams and their contribution to fetal growth, we studied the intake of radioactive deoxyglucose to maternal and fetal tissues at E17.5. In normoxic and hypoxic dams, the kidney had the highest glucose intake, followed by placenta and liver (Fig. 5a). Despite the high renal glucose intake, no glucosuria was detected in normoxic or hypoxic dams (Table 1). However slight proteinuria was present in both groups, its level being higher in the normoxic dams (Table 1). The most prominent difference between the groups in glucose intake to maternal tissues was seen in the heart, whose levels in the hypoxic dams approximated those in the brain whereas in normoxia, brain glucose intake exceeded that of the heart (Fig. 5a). The tissues with the next highest glucose uptake levels in both groups were pancreas, skeletal muscle and gonadal WAT (Fig. 5a). In accord with the enhanced lipolytic metabolism seen in late pregnancy, the intake of glucose to WAT was minimal in both groups, but even lower in the hypoxia dams (Fig. 5a). In general, for most tissues glucose intake was ~2-fold higher in the hypoxic compared with normoxic dams (Fig. 5a). Importantly, there were significant negative correlations between maternal renal or hepatic glucose intake and embryonic weight (r = −0.52, P < 0.05 and r = −0.55, P < 0.05, respectively).

To understand the molecular mechanisms contributing to the differences in glucose intake between hypoxic and normoxic dams, we determined the tissue mRNA levels for several key metabolic HIF target genes^8^. Of these mRNA for *Phosphofructokinase* (*Pfk)*, a HIF1-target gene and the key rate-limiting enzyme of glycolysis, was significantly upregulated in hypoxia at E17.5 in maternal liver, gonadal WAT and skeletal muscle (Fig. 5b–d), and in embryonic liver and embryonic skeletal muscle at E17.5 (Fig. 5e,f). In liver, the mRNA for *Insulin receptor substrate 2* (*Irs2*), a HIF2-target and a key protein for increasing insulin sensitivity^19^, was significantly upregulated in the hypoxic dams (Fig. 5b). The mRNA for an additional key HIF target gene central for downregulation of OXPHOS, *Pyruvate dehydrogenase kinase 1* (*Pdk1*), was increased in maternal gonadal WAT under hypoxia (Fig. 5c). Altogether, these analyses supported activation of glucose intake and glycolytic metabolism, and downregulation of OXPHOS in hypoxia dams.

## Discussion

The chronic hypoxia of residence at high altitude (>2500 m) lowers birth weight by asymmetric intrauterine growth restriction (IUGR), resulting in a greater reduction in birth weight than infant length^1–3^. Next to gestational age, high altitude has the greatest influence in birth weight, exceeding the influences, for example, of prenatal care or maternal smoking^1,20,21^. This is significant because IUGR not only raises infant mortality and morbidity but also increases susceptibility to cardiovascular and metabolic diseases later in life^13^. Thus identifying the mechanisms responsible for hypoxia-associated IUGR offers a novel means for identifying new treatments and preventive measures not only for the 140 million high-altitude residents worldwide^22^ but also for other cases of hypoxia-associated IUGR. Maternal physiological responses to pregnancy are little affected by the duration of the woman’s own residence at high altitude^23^ but the effects of immediate exposure to high altitude on pregnancy responses are unknown.

One of the key adaptations under hypoxia is to adjust energy metabolism so as to accord with the reduced supply of oxygen^4,8,24^. It is therefore important that the hypoxia-sensitive HIF pathway’s target genes regulate glucose and lipid metabolism. In general hypoxia and the HIF target genes promote non-oxygen demanding glycolytic metabolism while suppressing OXHOS^4,8,24^. A key mechanism for this is the upregulation of *PDK* that prevents pyruvate conversion to acetyl-CoA by pyruvate dehydrogenase and its further use for energy generation in the Krebs cycle or as a building block for lipogenesis^8^. HIF also promotes glucose intake to cells thus providing more substrate for glycolysis^8^. This is an attempt to compensate for the reduced energy balance because glycolysis generates significantly less ATP compared to OXPHOS. Under hypoxia the main end product of glycolysis is lactate. Its build-up acidifies cells and therefore HIF-targeted genes also promote lactate clearance and its reuse in gluconeogenesis and Cori cycle^25,26^.

**Figure 6:**
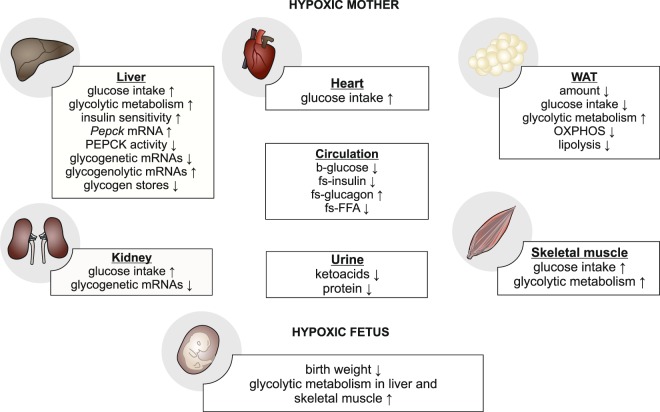
Schematic illustration of the maternal metabolic changes during pregnancy that contributed to reduced birth weight under hypoxia compared with normoxia. FFA = free fatty acids, OXPHOS = oxidative phosphorylation, Pepck = phosphoenolpyruvate carboxykinase.

Our data here show that under hypoxia the pregnant dams failed to increase the amount of adipose tissue during the anabolic phase of gestation to as great an extent as in the normoxic dams. This is likely due to the relative inefficiency of glucose metabolism under conditions of hypoxia, which in WAT is characterized by upregulation of the glycolytic metabolism inducing and OXPHOS suppressing *Pfk* and *Pdk1* mRNAs, respectively^8,15^ (Fig. 6). As the amount of gonadal WAT correlated positively with birth weight, the failure in lipogenesis appears to be a contributing factor to the altitude-associated IUGR.

Although insulin levels doubled in hypoxic dams in late pregnancy compared with mid gestation, their insulin resistance score did not change whereas the normoxic dams’ HOMA-IR score increased nearly 3-fold in late- compared with mid-gestation. The correlation between increased maternal glucose intake with reduced birth weight is of importance and suggests that improved maternal glucose tolerance has a deleterious effect on fetal growth or birth weight under hypoxia. One of the key metabolic adaptations during pregnancy is the increased utilization of fatty acids and decreased use of glucose for fueling cardiac metabolism^27^. The hypoxic dams appeared to fail in this as they took in ~2-fold more glucose to heart compared with normoxic dams. Altogether these data suggest that the hypoxic dams failed to generate the physiological insulin resistance necessary for supporting fetal growth especially in the last trimester (Fig. 6).

The effect of high altitude on IUGR is only half as great in multigenerational populations (Andeans, Tibetans) compared with shorter-resident groups (Europeans, Han Chinese)^22^. We and others have shown that multiple HIF-pathway genes have been acted upon by natural selection in multigenerational high-altitude populations and are associated with, for example, a lesser altitude-associated rise in Hb levels or reduction in birth weight at high altitude^28–32^. *PRKAA1*, coding for the catalytic subunit of adenosine monophosphate kinase (AMPK) that is a central regulator of cellular energy metabolism, has been acted upon by natural selection in Andeans with regards to birth weight under hypoxia^28,29^. Interestingly, several effects of AMPK and HIF on glucose and lipid metabolism are similar, and activation of both increase insulin sensitivity.

Whereas increased insulin sensitivity in well-fed Western populations can be considered favourable for health, our data suggest that the further rise in insulin sensitivity seen during hypoxic pregnancy results in greater glucose intake by maternal tissues and thereby limits glucose consumption for fetal growth. Based on our data, a high calorie diet, especially during the first two trimesters, might be an intervention to induce lipogenesis and ensure glucose supply during late gestation in high-altitude or otherwise hypoxic pregnancies. Moreover, modulation of insulin sensitivity with HIF or AMPK-targeting substances may be a way to direct more glucose for fetal growth in hypoxia. Viewed differently, our data suggest that exposure to high altitude or intermittent hypoxia may be treatment options for gestational diabetes characterized by impaired gestational glucose tolerance or for obese mothers where hyperinsulinemia has been identified as a key programming factor affecting the long-term health of offspring^33^.

In conclusion, exposure to environmental hypoxia during gestation reduced birth weight, maternal weight gain and the amount of maternal WAT in C57BL6/NCrl dams compared with normoxia housed dams. The reduced maternal adiposity under hypoxia contributed to impaired catabolism in late pregnancy evidenced *e*.*g*. by lower levels of urine ketoacis. The hypoxic dams had improved glucose tolerance and insulin sensitivity and elevated serum glucagon levels compared with normoxic dams. The increased uptake of glucose to maternal tissues under hypoxia correlated with upregulation of HIF-pathway genes. Altogether, these data suggest that interventions that can antagonize such changes in maternal metabolism may help to preserve fetal growth in high-altitude.

## Methods

### Animals and hypoxic intervention

All experiments were conducted according to the Finnish Act on Animal Experimentation (62/2006) and approved by National Animal Experiment Board of Finland. Young (3–6 months old) female C57BL/6NCrl mice were mated under normoxic conditions (21% oxygen) overnight. The next morning (E0.5) the mice were placed either in a hypoxic chamber (Hypoxic Glove Box, Coy Laboratory Products, USA) under 15% normobaric oxygen concentration, or kept under normoxia. The dams were weighed five days a week and fed *ad libitum* with Teklad Global Rodent diet T.2018C.12 (Harlan Teklad, USA) during gestation.

The dams were sacrificed using CO_2_ and embryos by decapitation at E9.5 or E17.5. Terminal blood and tissues were collected. Gonadal WAT, liver and placentas were weighed and tissues were snap frozen in liquid nitrogen. The embryo number was calculated, embryos stripped of fetal membranes, and the pooled embryo weight measured for each dam.

### Determination of blood hemoglobin, lactate, serum lipids, fatty acids and ketoacid levels

The blood hemoglobin was measured spectrophotometrically with HemoCue Hb 201 Analyzer. Blood lactate was measured using enzymatic-amperometric detection (Lactate Scout+ -meter;SensLab/EKF Diagnostics). The serum fraction was collected from the terminal blood of the dams at sacrifice. Serum total cholesterol and triglyceride levels were determined by an enzymatic method (Roche Diagnostics). Fatty acids were determined with the fluorometric Free Fatty Acid Quantification Kit (abcam #ab65341) and ketoacids with β-Hydroxybutyrate Assay Kit (Sigma Aldrich #MAK041).

### Histological analyses

WAT samples were fixed in formalin (10%) and embedded in paraffin. 5 µm sections of samples were cut and stained with hematoxylin-eosin, viewed and photographed with a Leica DM LB2 microscope and a Leica DFC 320 camera. Five representative pictures were taken per sample, and 50 adipocytes were quantified using Adobe Photoshop CS5 Magnetic Lasso Tool. The data were transferred to Microsoft Excel and analyzed.

### Urine analyses

The mouse urine was collected and analyzed for pH, ketoacids, glucose and protein with Combur-Test urinary test strips (Roche Diagnostics) according to the manufacturer’s instructions.

### Glucose tolerance test (GTT), determination of serum insulin and glucagon levels and calculation of HOMA-IR scores

GTT was performed on dams after a 4 h fast at E9.5 and 17.5. Mice were anesthetized with fentanyl/fluanisone and midazolam, and injected intraperitoneally with glucose (1 mg/g at E9.5 and 2 mg/g at E17.5). Blood glucose concentrations were monitored with a glucometer. Serum insulin and glucagon values were determined enzymatically with Rat/Mouse Insulin ELISA kit (Millipore #EZRMI-13K) and Mouse Glucagon ELISA Kit (CrystalChem #81518). HOMA-IR scores were calculated from the fasted glucose and insulin values ((fasting insulin (pmol/l) × fasting glucose (mmol/l))/156.65).

### Determination of PEPCK activity

The livers of pregnant dams, terminally anesthetized with fentanyl/fluanisone and midazolam, at E17.5 were dissected and a 50 mg sample was taken from the superior lobe before mice were sacrificed. The samples kept on ice were homogenized, the homogenate was centrifuged for 20 min at 4 °C, and the supernatant was collected. Supernatant was incubated for 10 min in 30 °C in a solution containing imidazole, DTE, NaF, KCl and MnCl_2_. The reaction was stopped by adding KBH_4_ and neutralized with HClO_4_ and KHCO_3_. The samples were centrifuged and their PEPCK activity was measured with an Aminco DW-dual Wavelength spectrophotometer at 366 nm^34^.

### Determination of glycogen

The pregnant dams were sacrificed at E17.5 after a 4 h fast with CO_2_, and 150 mg samples were taken from superior lobe of the liver, kidney and skeletal muscle (*M*. *quadriceps femoris*). Samples were snap frozen in liquid nitrogen and stored at −70 °C before analyzing with a fluorometric Glycogen Assay Kit (Cayman Chemical #700480).

### Quantitative real-time PCR (qPCR) analyses

Total RNA from the tissues was isolated with E.Z.N.A. Total RNA Kit II (Omega Bio-Tek) or TriPure Isolation Reagent (Roche Applied Science) and purified with E.Z.N.A. Total RNA Kit I (Omega Bio-Tek). Reverse transcription was performed with qScript cDNA Synthesis Kit (Quanta Bioscienses). Quantitative PCR was conducted with iTaq SYBR green Supermix with ROX (Bio-Rad) in a C1000 Touch Thermal Cycler and CFX96 Touch Real-Time PCR Detection System (Bio-Rad) with the primers shown in Table 2.

**Table 2:** Primer sequences for real-time quantitative PCR.

Gene	Forward primer	Reverse primer
*Bactin*	AGAGGGAAATCGTGCGTGAC	CAATAGTGATGACCTGGCCGT
*Gbe1*	ACTGCTTTGATGGCTTCCGT	AACCTTGACCCATTCCGTGG
*G6pc*	CGACTCGCTATCTCCAAGTGA	GTTGAACCAGTCTCCGACCA
*Gyg*	GCTGGTCACTTACTCAGTATTCC	AGGGTTGATAGACAAAGACTCCA
*Irs2*	GTAGTTCAGGTCGCCTCTGC	TTGGGACCACCACTCCTAAG
*Pdk1*	Quantitect primer assays (Qiagen)	
*Pepck1*	GAGGCCACAGCTGCTGCAGAA	GAAGAAGGGTCGCATGGCAAA
*Pfk*	Quantitect primer assays (Qiagen)	
*Ppia*	GAGCTGTTTGCAGACAAAGTTC	CCCTGGCACATGAATCCTGG

### Deoxyglucose uptake test

Deoxyglucose uptake test was performed at E17.5 after a 4 h fast. Mice were injected intraperitoneally with ^14^C-deoxyglucose (0.6 µCi/g) with glucose (1 mg/g) and sacrificed after 60 min. 50 mg tissue samples were collected and homogenized with chloroform:methanol (1:1) solution (or in case of WAT 2:1) and centrifuged. The pellet was re-extracted and the supernatants were combined and scintillated for ^14^C activity.

### Statistical analyses

Student’s two-tailed t tests were used for comparisons between the groups. For average embryo weight (Fig. 1c) and its ratio to placental weight (Fig. 1d), for which the direction of the comparison was specified in advance, a one-tailed test was employed. Areas under the curve (AUC) were calculated by the summary measures method. Pearson’s correlation coefficient was calculated to compare linear dependences between two variables. Values of ±3 SD were omitted from the statistical analyses. For HOMA-IR (Fig. 3d), for which the values did not follow a normal distribution log transformed values were used to calculate the P values. All data are mean ± SEM. P < 0.05 was considered statistically significant.
