# Author Correction: Hypoxia causes reductions in birth weight by altering maternal glucose and lipid metabolism

**DOI:** 10.1038/s41598-020-61220-x

**Published:** 2020-03-03

**Authors:** Jenni Määttä, Niina Sissala, Elitsa Y. Dimova, Raisa Serpi, Lorna G. Moore, Peppi Koivunen

**Affiliations:** 1 Biocenter Oulu, Faculty of Biochemistry and Molecular Medicine, Oulu Center for Cell-Matrix Research, University of Oulu, Oulu, Finland; 2 Department of Obstetrics & Gynecology, University of Colorado Denver School of Medicine, Aurora, CO United States

Correction to: *Scientific Reports* 10.1038/s41598-018-31908-2, published online 11 September 2018

This Article contains errors. In Figure 2b and 2c, the scales of the y-axes are incorrect. The correct Figure 2 appears below as Figure 1.

**Figure 1:**
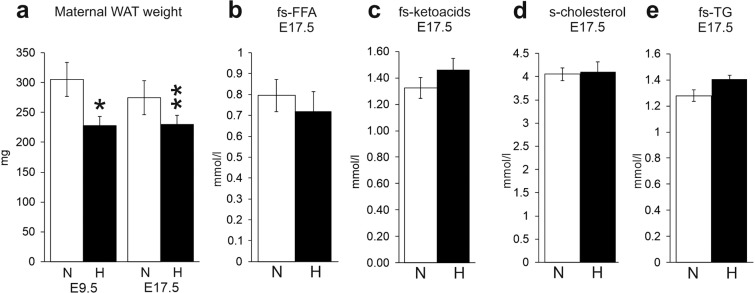
.

Additionally, in Figure 4a and 4d, the units of the y-axes are incorrect. The correct Figure 4 appears below as Figure 2.

**Figure 2:**
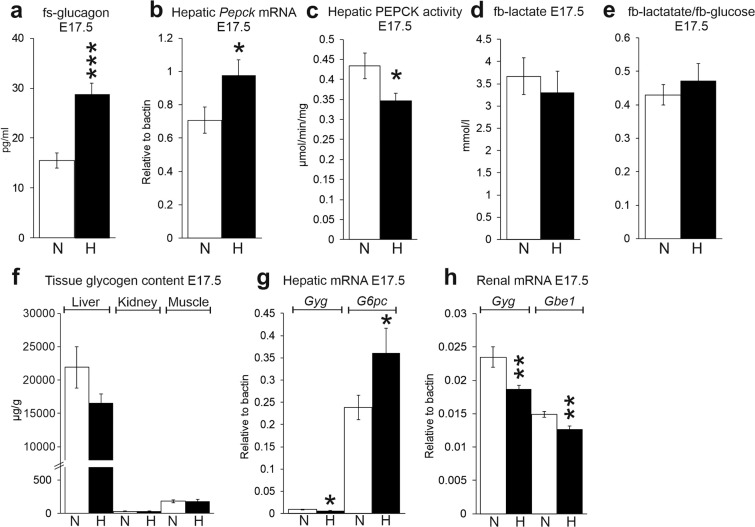
.

